# Detection of Cerebrovascular Loss in the Normal Aging C57BL/6 Mouse Brain Using *in vivo* Contrast-Enhanced Magnetic Resonance Angiography

**DOI:** 10.3389/fnagi.2020.585218

**Published:** 2020-10-20

**Authors:** Lindsay K. Hill, Dung Minh Hoang, Luis A. Chiriboga, Thomas Wisniewski, Martin J. Sadowski, Youssef Z. Wadghiri

**Affiliations:** ^1^Department of Chemical and Biomolecular Engineering, NYU Tandon School of Engineering, Brooklyn, NY, United States; ^2^Department of Radiology, Center for Advanced Imaging Innovation and Research (CAI2R), NYU Grossman School of Medicine, New York, NY, United States; ^3^Department of Radiology, Bernard and Irene Schwartz Center for Biomedical Imaging, NYU Grossman School of Medicine, New York, NY, United States; ^4^Department of Biomedical Engineering, SUNY Downstate Medical Center, Brooklyn, NY, United States; ^5^Department of Pathology, NYU Grossman School of Medicine, New York, NY, United States; ^6^Department of Neurology, NYU Grossman School of Medicine, New York, NY, United States; ^7^Department of Psychiatry, NYU Grossman School of Medicine, New York, NY, United States; ^8^Department of Biochemistry and Molecular Pharmacology, NYU Grossman School of Medicine, New York, NY, United States

**Keywords:** magnetic resonance (MR) angiography, blood pool agent, mouse brain aging, cerebral blood volume (CBV), rarefaction, MRI

## Abstract

Microvascular rarefaction, or the decrease in vascular density, has been described in the cerebrovasculature of aging humans, rats, and, more recently, mice in the presence and absence of age-dependent diseases. Given the wide use of mice in modeling age-dependent human diseases of the cerebrovasculature, visualization, and quantification of the global murine cerebrovasculature is necessary for establishing the baseline changes that occur with aging. To provide *in vivo* whole-brain imaging of the cerebrovasculature in aging C57BL/6 mice longitudinally, contrast-enhanced magnetic resonance angiography (CE-MRA) was employed using a house-made gadolinium-bearing micellar blood pool agent. Enhancement in the vascular space permitted quantification of the detectable, or apparent, cerebral blood volume (aCBV), which was analyzed over 2 years of aging and compared to histological analysis of the cerebrovascular density. A significant loss in the aCBV was detected by CE-MRA over the aging period. Histological analysis *via* vessel-probing immunohistochemistry confirmed a significant loss in the cerebrovascular density over the same 2-year aging period, validating the CE-MRA findings. While these techniques use widely different methods of assessment and spatial resolutions, their comparable findings in detected vascular loss corroborate the growing body of literature describing vascular rarefaction aging. These findings suggest that such age-dependent changes can contribute to cerebrovascular and neurodegenerative diseases, which are modeled using wild-type and transgenic laboratory rodents.

## Introduction

Pre-clinical mouse models continue to improve our understanding of clinically relevant biological and pathological processes and aid in our ability to diagnose and treat human diseases. The use of mice in biological research has led to substantial insight into their anatomy and, more recently, into murine cerebrovascular architecture. The cerebrovasculature is critical in supplying the brain with oxygen and nutrients to maintain normal brain function and cognition (Zlokovic, 2011; Hirsch et al., 2012; Ungvari and Sonntag, 2014). Alterations to the cerebrovasculature contribute to brain pathologies including brain tumors, stroke and transient ischemic attack, vascular dementia, Alzheimer’s disease, and leukoaraiosis (de la Torre, 2004; Brown and Thore, 2011; Hirsch et al., 2012; Ni et al., 2019). Age-dependent reductions in the cerebrovascular density, or rarefaction (Bullitt et al., 2010), have been shown to contribute to reduced cerebral blood flow and perfusion, resulting in impaired clearance of misfolding-prone proteins and peptides that constitute the premise for several neurodegenerative diseases, including Alzheimer’s disease (Martin et al., 1991; Klohs et al., 2014; Tarasoff-Conway et al., 2015; Yang et al., 2017). Notably, age is one of the most important independent risk factors in such vascular diseases (Brown and Thore, 2011; Hirsch et al., 2012; Xu et al., 2017). Therefore, our understanding of the murine cerebrovasculature and how it is impacted by normal aging is critical in studying age-dependent diseases of the brain.

In addition to well-characterized region-dependent changes in brain volume with age (Walhovd et al., 2005; Lockhart and DeCarli, 2014), aging studies of the cerebrovasculature, conducted primarily in humans and rats, have largely concluded that microvascular rarefaction occurs in the normal aging brain (Riddle et al., 2003; Brown and Thore, 2011). The extent of rarefaction is variable and region-dependent (Brown and Thore, 2011); for example, with capillary reductions reported in aged rats from 12% (Burns et al., 1981) to 43% (Amenta et al., 1995) in the cerebral cortex and from 20% (Jucker et al., 1990) to 49% (Amenta et al., 1995) in the hippocampus. Recently, *ex vivo* confocal microscopy of sectioned brains from aged C57BL/6 mice demonstrated region-dependent capillary density reductions from 19% to 34.5% over 2 years (Murugesan et al., 2012). While in agreement with prior human and rat studies, whole-brain quantifications are required to appreciate the impact of aging on the global murine cerebrovasculature.

Until recently, much of our understanding of the cerebrovasculature was contributed by *ex vivo* 2D histology (Hirsch et al., 2012; Wu et al., 2014), still considered the gold standard for visualizing tissue microvasculature (Moy et al., 2013). Recent advances have aimed to observe cerebrovasculature throughout the whole murine brain with high-resolution, including *via* micro-CT with or without vascular corrosion casting (Krucker et al., 2004, 2006; Heinzer et al., 2006, 2008; Dorr et al., 2007; Meyer et al., 2008; Chugh et al., 2009; Ghanavati et al., 2014a,b), scanning electron microscopy (Krucker et al., 2004, 2006), micro-optical sectioning tomography (MOST; Li et al., 2010; Wu et al., 2014; Xue et al., 2014; Xiong et al., 2017), and CLARITY (Chung et al., 2013; Zhang et al., 2018). While these techniques provide images with impressively high spatial resolution, they are only achievable *ex vivo* and are often destructive or otherwise induce tissue deformation or shrinkage (Wehrl et al., 2015). High-resolution *in vivo* techniques, such as two-photon microscopy, have also been used to quantify the cerebral blood volume (CBV), but the technique is largely limited to evaluating the cortical vasculature due to limited tissue penetrance (Serduc et al., 2006; Steinman et al., 2017). Still, such techniques have been employed to quantify the murine cerebrovasculature, which was found to range from 1% to 4.4% of the total brain volume (Boero et al., 1999; Heinzer et al., 2006, 2008; Serduc et al., 2006; Chugh et al., 2009; Tsai et al., 2009; Wu et al., 2014; Zhang et al., 2018). However, given the dynamic and often heterogeneous process of aging and age-dependent diseases, studies on changes in CBV or microvascular density would benefit from *in vivo* techniques, enabling individual subjects to be studied longitudinally.

Magnetic resonance angiography (MRA) is a non-destructive 3D imaging technique that can achieve repeatable *in vivo* neuroimaging of the cerebrovascular network (Nishimura et al., 1986; Beckmann, 2000; Krucker et al., 2004). Traditional time of flight (TOF)-MRA relies on fast laminar blood flow within the vascular space of proximal in-flowing arteries and, therefore, lacks signal in slow-flowing veins and tortuous and distal vessels (Axel, 1984; Nishimura, 1990). Contrast-enhanced MRA (CE-MRA), however, provides cerebrovascular signal indiscriminate of vessel size, flow, and location within the field of view due to the improved relaxation of water by an exogenous *T*_1_-shortening contrast agent (Howles et al., 2009). Traditional small molecule *T*_1_-agents, such as gadolinium-diethylenetriamine pentaacetic acid (Gd-DTPA, Magnevist), have long been used for clinical CE-MRA acquisition (Lohrke et al., 2016), but their small size allows for extravasation from the porous vasculature outside of the brain into the interstitial space, resulting in a lack of steady-state signal enhancement (Estelrich et al., 2015). This leakage is particularly deleterious for small animal imaging, which must not only consider the 4.5–6-fold shorter plasma half-lives for such agents in rodents (Aime and Caravan, 2009) but also requires considerably longer imaging times than clinical acquisitions for comparable anatomical resolution (Driehuys et al., 2008). To avoid vascular extravasation, blood pool contrast agents have been employed due to their larger size, a result of serum protein binding or multimeric macromolecular design (Caravan et al., 1999; Torchilin, 2000; Torchilin et al., 2000; Lee et al., 2012; Nielsen and Thomsen, 2012), thus slowing their rate of clearance and permitting the use of a longer imaging time (Estelrich et al., 2015) needed for higher spatial resolution (Nielsen and Thomsen, 2012).

In this study, we have employed Gd-micelle CE-MRA to quantify the cerebrovascular density in aged C57BL/6 mice, the most widely used inbred mouse strain (Bryant, 2011), throughout a 2-year aging period. Mice were imaged *via* CE-MRA at 2–4 months (young adulthood), 14–16 months, and 24–26 months (aged adulthood). A subset of mice was studied longitudinally, allowing for the tracking of individuals throughout the 2-year study, while another subset was used for immunohistochemical (IHC) analysis of the cerebrovasculature. To achieve high signal intensity within the cerebrovascular space, we synthesized a lipid-based Gd-DTPA-bearing micelle, based on a modified version of the micellar design reported by Briley-Saebo et al. (2006, 2008). MRA datasets were aligned using automated registration (Friedel et al., 2014) allowing for virtual whole brain and regional segmentation (Dorr et al., 2008). The detected CBV within segmented regions, here dubbed the apparent CBV (aCBV), was quantified and compared between age groups along with the whole brain and ventricular volumetric assessment. The gold standard technique for microvascular visualization, IHC, was employed for comparative quantification of the microvascular density (McDonald and Choyke, 2003; Moy et al., 2013). While CE-MRA of the whole mouse head is limited in spatial resolution compared to IHC and other *ex vivo* techniques, it provides *in vivo* cerebrovascular information with whole brain coverage. Together, we have employed these techniques to quantify changes within the cerebrovascular density of normal aging wild-type C57BL/6 mice in an effort to elucidate vascular alterations due to aging that contribute to age-dependent diseases of the cerebrovasculature.

## Materials and Methods

### Materials

Three lipids, 1,2-distearoyl-sn-glycero-3-phosphoethanolamine-N-[methoxy(polyethylene glycol)-2000] (ammonium salt) (PEG-2000-DPSE), DTPA-bis(stearylamide) (gadolinium salt) (Gd-DTPA-bis(stearylamide)), and 1,2-dipalmitoyl-sn-glycero-3-phosphoethanolamine-N-(lissamine rhodamine B sulfonyl) (ammonium salt) (Rhodamine-DPPE) were purchased from Avanti Polar Lipids. Chloroform, methanol, and [4-(2-hydroxyethyl)-1-piperazineethanesulfonic acid] (HEPES) were purchased from Sigma-Aldrich. Sodium chloride was from Thermo Fisher Scientific. Ethylenediaminetetraacetic acid was from Acros Organics. Acrodisc syringe filters, 0.2 μm, for micelle filtration were purchased from PALL. Magnevist (gadopentetate dimeglumine) was from Bayer Corporation. Intramedic^TM^ Clay Adams^TM^ brand polyethylene PE-10 tubing (inner diameter 0.28 mm, outer diameter 0.61 mm) was purchased from Becton Dickinson for cannulation and contrast agent injection. Vetbond^TM^ was purchased from the 3M Company. Isoflurane was purchased from Piramal Enterprises and Ketathesia (ketamine HCl injection) was from Henry Schein Animal Health.

### Micelle Synthesis

Gd-micelles were synthesized using a thin-film protocol modified from Briley-Saebo et al. (2008). Three synthetic phospholipids, PEG2000-DSPE, DTPA-bis(stearyl amide), and Rhodamine-DPPE (Avanti Polar Lipids, Alabaster, AL, USA), were combined (molar ratio 450:500:1) and dissolved in a 100:2 chloroform:methanol solution. A thin lipid film was generated under heat, at 68°C, and vacuum on a rotary evaporator (RV10, IKA-Werke, Staufen im Breisgau, Germany). Gd-micelles were formed over 20 min on the evaporator by rehydrating the film in HEPES buffer, pH 7.0, under gentle agitation at 65°C in the absence of a vacuum. The hydrated product was filtered with a 0.2 μm syringe filter before characterization.

### Micelle Characterization

The micellar hydrodynamic diameter was measured using dynamic light scattering (DLS; Zetasizer Nano Series model Nano ZS90, Malvern Instruments, Malvern, UK) in a low volume disposable cuvette. A 50 μl aliquot of filtered micelles was added to 700 μl of HEPES and measurements were taken in triplicate, conducting 10 runs for each measurement (5 s per run). The Gd concentration within the micelles was determined *via* inductively coupled plasma-optical emission spectrometry (ICP-OES) by Galbraith Laboratories, Inc. (Knoxville, TN, USA). The longitudinal relaxivity, *r*_1_, and the transverse relaxivity, *r*_2_, of HEPES-diluted Magnevist (Gd-DTPA) and Gd-micelles were determined at 40°C on a 60 MHz (1.4-Tesla) Bruker minispec mq-one TD-NMR (Bruker Biospin, Billerica, MA, USA) using a previously described protocol (Briley-Saebo et al., 2006, 2008). Longitudinal and transverse relaxation times, *T*_1_ and *T*_2_, respectively, were acquired for six different concentrations of contrast agent diluted from a concentration of 3 mM Gd. *T*_1_ values were determined using an inversion recovery sequence with 15 inversion times from 10 to 1,000 ms and *T*_2_ values were acquired using a Car-Purcell-Meiboom-Gill (CPMG) spin-echo sequence where the inter-echo time varied between 0.1 and 2.0 ms. The inverse of the relaxation times *T*_1_ and *T*_2_ were calculated to obtain the corresponding relaxation rates, *R_1_* and *R*_2_. Relaxivity values, *r*_1_ and *r*_2_, were separately calculated as the slopes of Gd concentration vs. relaxation rate, *R*_1_ or *R*_2_.

### Animals

All mouse care and experimental procedures were approved by the Institutional Animal Care and Use Committee of the New York University School of Medicine. All *in vivo* MRA experiments were performed on female C57BL/6 wild type mice. The C57BL/6 strain was chosen due to its popularity and importance in biomedical research (Bryant, 2011). Only female mice were studied to minimize effects and variability due to sex (Murugesan et al., 2012). Furthermore, the aging of male C57BL/6 mice often necessitates individual housing for each subject due to increased aggression with age (Svare et al., 1983; Eskola and Kaliste-Korhonen, 1999; An et al., 2011), which is considerably more costly than group-housing aging female mice. C57BL/6NTac mice purchased from Taconic Biosciences (Rensselaer, NY, USA) were used for micelle dosage studies at 2–4 months old and for longitudinal high-resolution MRA studies at 2–4 months (*N* = 5), 14–16 months (*N* = 5), and 24–26 months (*N* = 3, as two subjects died before the final imaging session) following aging in-house. C57BL/6N mice at 2–4 months (*N* = 9) were subsequently purchased from Charles River Laboratories (Wilmington, MA, USA) for additional MRA studies and histological analysis. Aged C57BL/6N mice were also acquired from National Institute on Aging, housed at Charles River Laboratories, at 14–16 months (*N* = 7) and 24–26 months (*N* = 5) for additional MRA studies and histological analysis at these age groups.

### Femoral Vein Cannulation and Animal Setup

Before each imaging session, mice were anesthetized *via* isoflurane inhalation at 1.5 L min^−1^ oxygen using a vaporizer/anesthesia setup (VetEquip, Inc., Livermore, CA, USA). Up to 5% isoflurane in the air was used for anesthesia induction, followed by 1.0–1.5% isoflurane in the air *via* nose cone throughout the femoral vein cannulation procedure. Following anesthetic induction, mice were placed in the supine position on an electric heating blanket to maintain body temperature between 35 to 37°C. Fur over the femoral area of the hind limb was removed after the 30-s application of Nair^®^ hair removal cream (Church and Dwight Company, Inc., Ewing Township, NJ, USA) using sterile cotton swabs. The area was rinsed with sterile water until all cream was removed. The surgical area was sterilized with Betadine and a 70% ethanol scrub. A 0.5 cm incision was made in the surgically prepared area over the femoral vein. If necessary, sterile blunt-tipped scissors were employed to gently remove connective tissue until the femoral vein was exposed. A 31-gauge needle was used to make a small incision in the vein. Contrast agent-primed PE-10 polyethylene tubing (Intramedic, Becton Dickinson, Franklin Lakes, NJ, USA), previously thinned using heat, was attached to a contrast agent-filled syringe and inserted approximately 0.3 cm into the femoral vein. The cannula was held in place with Vetbond^TM^ (3M Company, Maplewood, MN, USA) and allowed to dry. Once dry, mice were placed in the prone position with their heads restrained on a house-made 3D-printed imaging holder equipped with a bite bar and ear bars to minimize motion. The mouse head was inserted into a custom-made radiofrequency (RF) coil built in-house for MRI acquisition, while the rest of the subject’s body was covered with 3D-printed water-circulating warming pads to maintain a body temperature between 35 to 37°C. The whole mouse bed was inserted into the center of the bore magnet. Body temperature and breathing rate were monitored continuously throughout the image acquisition (SA Instruments Inc., Stony Brook, NY, USA) and maintained with 1.0–1.5% isoflurane in the air *via* a nose cone. Respiratory motion was monitored using a pneumatic pillow fixed to the subject’s abdomen and the core body temperature was measured *via* a rectal probe. The length of PE-10 polyethylene cannula tubing used was long enough to enable remote contrast infusion using a PHD-2000 computer-controlled syringe pump (Harvard Apparatus, Holliston, MA, USA). With the mouse remaining in the magnet, the contrast agent was injected *via* a syringe pump at a 60 μl min^−1^ infusion rate. The image acquisition began after a 1 min circulation period. The cannula was removed following image acquisition and the skin was sutured. Mice were allowed to recover in a heated cage.

### MRA Acquisition

All MRA experiments were performed on a 7-Tesla (7-T) micro-MRI system consisting of a 7-T 200 mm horizontal bore magnet (Magnex Scientific Limited, Yarnton, UK) interfaced to a Bruker Biospec Avance-2 console (Bruker Biospin, Billerica, MA, USA). The system was equipped with an actively shielded gradient coil (Resonance Research, Billerica, MA, USA: BGA-9S; ID 90 mm, 750 mT m^−1^ gradient strength, 100 μs rise time). A circularly polarized RF probe was developed in-house to resonate at a proton frequency of 300 MHz in both transmit and receive modes. Probe dimensions (length = 29 mm, outer diameter = 23.5 mm, and accessible diameter = 21.5 mm) ensured homogenous RF coverage of the whole adult mouse head. A modified three-dimensional (3D) *T*_1_-weighted spoiled gradient recalled echo (SPGRE) sequence was employed to acquire an additional self-gated signal during the readout dephasing gradient within each repetition time (TR; Nieman et al., 2009). The gating signal was used retrospectively to correct for motion artifacts induced by respiration and generate artifact-free image reconstruction sets. Scan parameters were as follows: echo time, TE = 4.07 ms; TR = 50 ms; bandwidth, BW = 75 kHz; number of averages, NAV = 1; number of repetitions, NR = 3. The flip angle (FA) = 34° was chosen to provide the greatest *T*_1_-enhancement contrast (Neelavalli and Haacke, 2007). Only the field of view (FOV), matrix size, and imaging time (T_IM_) varied between low-resolution (150 μm)^3^ scans (FOV = 19.2 mm × 19.2 mm × 19.2 mm, matrix size = 128 × 128 × 68, *T*_IM_ = 30 min) and high-resolution (100 μm)^3^ scans (FOV = 25.6 mm × 25.6 mm × 25.6 mm, matrix size = 256 × 256 × 136, *T*_IM_ = 87 min). 3D imaging with isotropic resolution enables the image set to be reprocessed in any desired slice orientation, facilitating image comparison during co-registration between separately acquired subjects. The appropriate concentration of Gd-micelle administered for high-resolution (100 μm)^3^ MRA studies was determined by a time course study comprised of serial (150 μm)^3^ MRA acquisitions using varying Gd-micelle doses.

### Image Registration and Segmentation

Following (100 μm)^3^ MRA acquisition of mice in all age groups, angiograms were compiled for automated anatomical registration using the mouse-build-model (MBM) pipeline within the Pydpiper toolkit, developed at the Hospital for Sick Children’s Mouse Imaging Centre (Friedel et al., 2014). All images were compiled and aligned to a (100 μm)^3^-resolution modified C57BL/6J mouse brain anatomical atlas developed by Dorr et al. (2008) using the MBM pipeline. The whole brain and brain regions (cerebral cortex, cerebellar cortex, entorhinal cortex, hippocampus, and striatum) were segmented for each aligned image using command-line tools from minc-stuffs, a suite of Medical Imaging NetCDF (MINC) tools, for image analysis (Vincent et al., 2004, 2016). The cerebral cortex, hippocampus, and entorhinal cortex were chosen for examination as these regions have previously demonstrated vascular and volumetric changes in diseases including Alzheimer’s disease and vascular dementia (Schuff et al., 2001; Krucker et al., 2004; Kara et al., 2012), in addition to previously reported age-dependent microvascular rarefaction in the cortex and hippocampus (Riddle et al., 2003; Xu et al., 2017; Yang et al., 2017). The cerebellar cortex was assessed as it also demonstrates atrophy and vessel alterations with age, including small vessel disease, micro-infarcts, and venous ischemia (Hoogendam et al., 2012; Cerchiai et al., 2017; De Cocker et al., 2017). Lastly, the striatum was included as vascular changes and reduced blood flow in the aging striatum may contribute to vascular parkinsonism and Parkinson’s disease (Feekes and Cassell, 2006; Guan et al., 2013; Afonso-Oramas et al., 2014; Gray and Woulfe, 2015).

### Apparent Cerebral Blood Volume Quantification

Segmented brains and brain regions were imported into ImageJ software (National Institute of Health, Bethesda, MD, USA; Schneider et al., 2012), and the enhanced cerebrovasculature was assessed qualitatively by generating a Maximum Intensity Projection (MIP) using the 3D projection function. The MIP algorithm highlights voxels of the highest intensity from a 3D image set to be projected onto a 2D plane, aiding in the visualization of signal enhancing contrast agent distributed throughout the tissue. Quantitation of micelle-enhanced voxels corresponding to the aCBV was also performed in ImageJ *via* intensity-based segmentation using a thresholding function in which the aCBV was defined as the percentage of (100 μm)^3^ voxels, within all voxels examined, demonstrating a signal intensity (SI) defined by the equation below. The threshold value of 2.5-fold the standard deviation above the mean SI of the region was chosen because it was the minimum value providing vascular segmentation while minimizing the inclusion of apparent background tissue:

$$SI_{CBV}\geq Mean\;SI_{ROI}+2.5\cdot ST\;Dev$$

### Deformation-Based Morphometry

RMINC, a package developed by the Mouse Imaging Center to read and write MINC volumes within the R environment, was used to perform statistical analyses on volumetric differences in mice of different age groups (Lerch et al., 2008). A deformation field was calculated between individual mice and a common space set by the Dorr et al. (2008) mouse brain atlas. The Jacobian determinant of the deformation field measured the expansion or contraction of volumes of interest on a voxel-wise basis (Chung et al., 2001) and significant volume changes were calculated per voxel with a false discovery rate, FDR, of <5% (Genovese et al., 2002). Volume differences in the segmented whole brain and ventricular system (the compiled lateral ventricles, third ventricle, cerebral aqueduct, and fourth ventricle) were also calculated and assessed for significance using one-way analysis of variance (ANOVA) test and Tukey’s honestly significant difference (HSD) *post hoc* test for multiple pair-wise comparisons between the three age groups.

### Histological Preparation and Immunohistochemistry of the Cerebrovasculature

Following micelle clearance, i.e., approximately 24 h after image acquisition, five mice per age group (2–4, 14–16, and 24–26 months) were sacrificed for histological examination, which was conducted by the Experimental Pathology Research Laboratory at New York University School of Medicine. IHC against CD31 to detect vascular endothelium was performed on paraformaldehyde-fixed, paraffin-embedded, 5 μm murine brain sections. CD31, also known as a platelet-endothelial cell adhesion molecule (PECAM-1), is constitutively expressed by all endothelial cells and is, therefore, a widely used marker for vascular staining (Amtul and Hepburn, 2014). Immunofluorescence staining was performed on a Ventana Medical Systems Discovery XT instrument using Ventana’s reagents and detection kits unless otherwise noted. Tissue sections were deparaffinized online and antigen retrieved using cell conditioner 1 for 36 min. Endogenous peroxidase activity was blocked with hydrogen peroxide. Unconjugated rabbit anti-mouse CD31 monoclonal antibody [Platelet Endothelial Cell Adhesion Molecule (clone D8V9E), CST #77699, Cell Signaling Technology, Danvers, MA, USA] was diluted 1:200 in Cell Signaling diluent and applied to the brain tissue for 2 h at room temperature. Following extensive washing in Ventana reaction buffer, the binding of the primary antibody to CD31 was detected with pre-diluted Ventana horseradish peroxidase-conjugated anti-rabbit secondary antibody, which was applied for 16 min and subsequently visualized with pre-diluted Ventana tyramide-conjugated rhodamine for 8 min. Slides were washed in distilled water and coverslipped with Prolong Gold anti-fade media (Molecular Probes, Eugene, OR, USA). A 1.0 mm tissue microarray composed of paraformaldehyde-fixed, paraffin-embedded murine tissues (skin, lung, liver, kidney, and brain) served as positive controls for optimization. The tissue microarray was also used as a negative control by using Cell Signaling diluent only for the primary antibody incubation. Stained brains were imaged on a NanoZoomer whole-slide scanner (Hamamatsu Photonics, Shizuoka, Japan) and analyzed in Visiopharm software (Hoersholm, Denmark).

Visiopharm’s CD31-stained vessel quantification application was employed to detect vessels emitting fluorescence in the red channel (RGB-R) above a threshold that prevented the detection of background tissue. The density of vessels per brain section was counted and divided by the area of the section in mm^2^ to acquire the number of vessels/mm^2^ in 2–4, 14–16, and 24–26 months brains (*N* = 5 sections per age). The same method for vascular analysis was applied to specific cortical and hippocampal regions of interest (ROIs) within the brain sections.

### Statistical Analysis

GraphPad Prism (GraphPad Software, San Diego, CA, USA) was utilized for all statistical analyses, except for that of deformation-based morphometry, which was built into the RMIC package. Specific statistical tests used are defined below for individual experiments. Differences were deemed statistically significant when demonstrating *p* < 0.05 (*), *p* < 0.01 (**), *p* < 0.001 (***), or *p* < 0.0001 (****).

For analysis of the aCBV obtained *via* MRA, aCBV values in the whole brain and brain regions were tested for normality using the D’Agostino-Pearson omnibus test (D’Agostino, 1986), the Shapiro–Wilk test (Shapiro and Wilk, 1965), and the Kolmogorov–Smirnov test with Dallal-Wilkinson-Lillie for *P*-value (Kolmogorov, 1933; Smirnov, 1939). Datasets were considered of normal distribution if they passed two out of the three normality tests (Boutajangout et al., 2010). The aCBV results were subsequently compared and significance was assessed using a one-way ANOVA test and Tukey’s HSD *post hoc* test for multiple pairwise comparisons between the three age groups for the entire female C57BL/6 cohort [2–4 (*N* = 14), 14–16 (*N* = 12), and 24–26 months (*N* = 8)]. Additionally, the aCBV values of in-house longitudinally-aged and imaged female C57BL/6NTac mice [2–4 (*N* = 5), 14–16 (*N* = 5), and 24–26 months (*N* = 3)] and the C57BL/6N mice imaged at single time points [2–4 (*N* = 9), 14–16 (*N* = 7), and 24–26 months (*N* = 5)] were separately analyzed *via* one-way ANOVA and Tukey’s HSD test between age groups. Separately, unpaired two-tailed Student’s *t*-tests were conducted to determine if there was a significant difference in the aCBV values of the C57BL/6NTac and C57BL/6N sub-strain cohorts in each brain region studied.

For CD31-stained histological sections, the density of vessels in whole brain sections, as well as cortical and hippocampal ROIs, were compared for 2–4, 14–16, and 24–26 months brains *via* one-way ANOVA and Tukey’s HSD *post hoc* test. CD31-stained vessels were also stratified by diameter, *via* minor axis length, using Visiopharm software with cut-offs of <50 μm, 50–100 μm, and >100 μm. All quantifications were subjected to two-way ANOVA and Tukey’s HSD *post hoc* test to assess significance across age groups.

## Results

### Gd-Micelle Characterization as a *T*_1_-Shortening Blood Pool Agent

**Figure 1:**
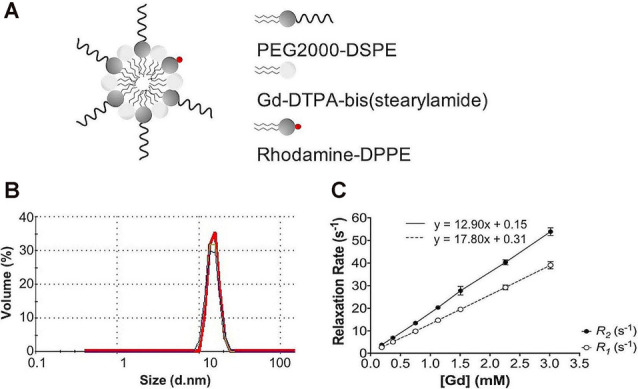
Gd-micelle composition and characterization. **(A)** Schematic of the Gd-micelle construct composed of three phospholipids: PEG2000-DSPE, Gd-DTPA-bis(stearyl amide), and Rhodamine-DPPE. **(B)** Hydrodynamic diameter determined by dynamic light scattering. **(C)** Longitudinal and transverse relaxation properties of Gd-micelle agent with error bars indicating the standard deviation amongst *N* = 5.

Following each batch synthesis, Gd-micelles (Figure 1A) were characterized for their hydrodynamic diameter, Gd concentration, and relaxation properties (Table 1, Figures 1B,C) to evaluate their capacity to serve as a blood pool agent with high relaxation properties. DLS confirmed an average hydrodynamic diameter of 15.63 ± 0.58 nm (*N* = 5) for >99.0% of the micelle population by volume, within the 10–2,000 nm range typical for blood pool agents (Torchilin et al., 2000; Table 1, Figure 1B). The Gd concentration, assessed commercially *via* ICP-OES (Galbraith Laboratories, Knoxville, TN, USA), was 11.70 ± 0.85 mM (*N* = 5; Table 1). The relaxation properties of Gd-micelles were compared to that of Magnevist at 60 MHz, corresponding to 1.4-Tesla, field strength (Table 1, Figure 1C). Specifically, the longitudinal and transverse relaxivity values, *r*_1_ and *r*_2_, were calculated as indicators of the MR signal enhancement or reduction potential, respectively (Burtea et al., 2008). The ratio *r*_2_/*r*_1_ was also calculated to assess of the agents’ suitability for positive (*T*_1_) or negative (*T*_2_) contrast (Burtea et al., 2008). Magnevist demonstrated an *r*_1_ of 3.29 mM^−1^ s^−1^ and *r*_2_ of 3.37 mM^−1^ s^−1^ at a temperature of 37^o^C, comparable to values previously described (Rohrer et al., 2005; Table 1). Its *r*_2_/*r*_1_ ratio of 1.02 confirms its use as a *T*_1_ agent, where 1.0 characterizes an optimal *T*_1_ shortening agent (Hashemi et al., 2004). Gd-micelles demonstrated an *r*_1_ of 12.90 ± 0.49 mM^−1^ s^−1^, 3.92-fold higher than the *r*_1_ value of Magnevist, and *r*_2_ of 17.80 ± 0.66 mM^−1^ s^−1^ (*N* = 5; Table 1, Figure 1C). The resulting *r*_2_/*r*_1_ was 1.38 ± 0.01, remaining within the range of effective *T*_1_-shortening contrast agents (*r*_2_/*r*_1_ = 1–2; Hagberg and Scheffler, 2013).

**Table 1:** Physicochemical properties of Gd-constructs.

Construct	Hydrodynamic diameter (nm ± STDev)	Gadolinium concentration (mM ± STDev)	*r*_1_ at 60 MHz in HEPES (mM^−1^ s^−1^ ± STDev)	*r*_2_ at 60 MHz in HEPES (mM^−1^ s^−1^ ± STDev)	*r*_2_/*r*_1_ at 60 MHz
Gd-micelle	15.63 ± 0.58	11.70 ± 0.85	12.90 ± 0.49	17.80 ± 0.66	1.38 ± 0.01
Gd-DTPA (Magnevist)	*	#x0003C;1 nm	*500	3.29	3.37	1.02

Characterization of the hydrodynamic diameter, Gd concentration, and relaxation properties at 60 MHz (1.4-Tesla) of Gd-micelle contrast agent compared to Magnevist. Gd-micelle data represent average values and standard deviations (N = 5). *Data determined by Bayer Corporation, Leverkusen, Germany.

### *In vivo* 3D CE-MRA of Whole Murine Brains

**Figure 2:**
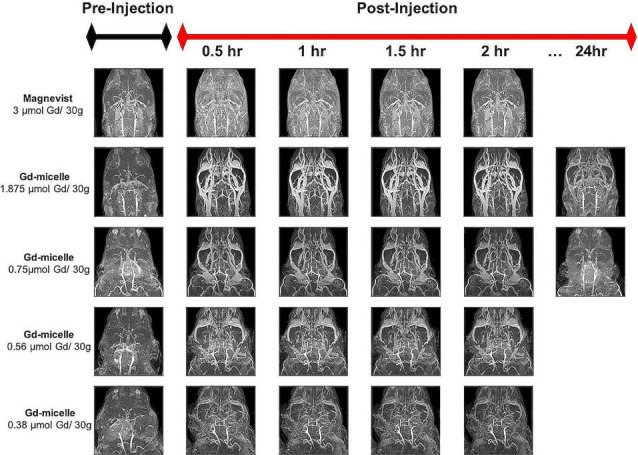
Time-course contrast-enhanced magnetic resonance angiography (CE-MRA) using Magnevist and Gd-micelle contrast. Time-course MRA study composed of a series of 30 min (150 μm)^3^ acquisitions pre- and post-injection of Magnevist, at the clinical equivalent dose by weight, or decreasing doses of Gd-micelle. Datasets are displayed as Maximum Intensity Projections (MIPs) to illustrate cerebrovascular enhancement.

Dosage studies compared the clinical equivalent dose of Magnevist by weight (3 μmol Gd/30 g) to varying doses of the Gd-micelle construct (from 0.38–1.875 μmol Gd/30 g) using 30 min (150 μm)^3^ *T*_1_–w angiograms in 2–4 months mice (Figure 2). Maximum intensity projections of the resulting angiograms demonstrated that Magnevist extravasated from the peripheral vasculature within 30 min, as determined by the enhanced signal intensity seen in the facial tissue of the mouse. As a result of this lack of steady-state recirculation, the signal enhancement in both the vasculature and the tissue returned to baseline within 2 h, suggesting that Magnevist would be inadequate as a steady-state *T*_1_ agent for high-resolution (100 μm)^3^ angiography. By contrast, Gd-micelles remained circulating within the vascular space as expected given the agent’s blood pool agent size (Torchilin et al., 2000) and PEGylation (Croy and Kwon, 2006; Nishiyama and Kataoka, 2006; Qiu et al., 2007). The dose of 0.75 μmol Gd/30 g was chosen for subsequent high-resolution MRA studies as it provided steady-state signal enhancement within the vascular space throughout the 2 h time-course, yet was cleared from circulation by 24 h, returning to the pre-injection baseline.

**Figure 3:**
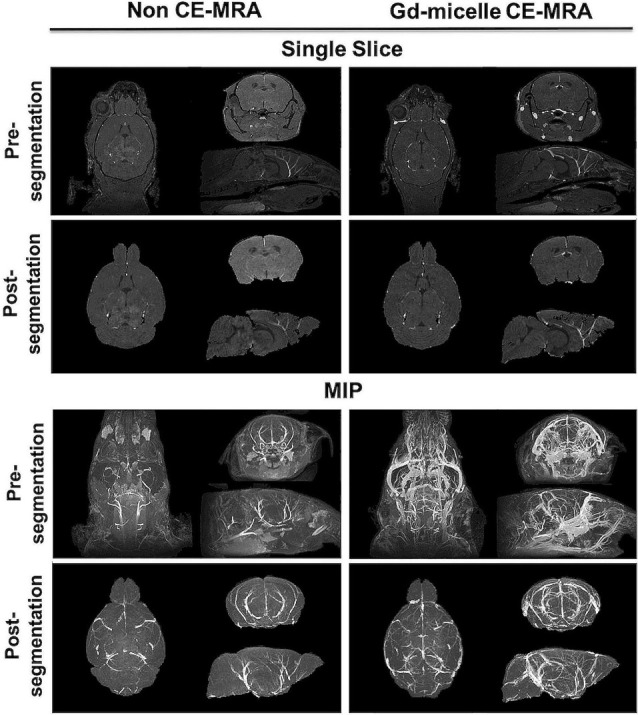
High-resolution MRA with and without Gd-micelle enhancement. 3D high-resolution MRA with and without Gd-micelle enhancement displayed as 2D single slices and MIPs pre- and post-virtual brain segmentation *via* registration to an anatomical brain atlas.

A (100 μm)^3^ *T*_1_-w MRA sequence was employed to test the impact of 0.75 μmol Gd/30 g Gd-micelle administration on the signal-to-noise ratio (SNR) of the murine vascular space. The same 2–4 months C57BL/6 mouse was imaged without and with Gd-micelle enhancement and the images were aligned for direct comparison of vessel enhancement (Figure 3). In the absence of contrast enhancement, the detectable vascular space demonstrated an endogenous SNR, averaged over 10 regions of interest (ROIs), of 36.14 ± 13.89. Following Gd-micelle administration at 0.75 μmol Gd/30 g, the resulting angiogram revealed an intravascular SNR of 115.13 ± 20.46, a 3.19-fold improvement. Notably, only vessels detectable in both angiograms, with and without contrast, were compared for signal enhancement, but many vessels were undetectable in the absence of Gd-micelle injection. As demonstrated by MIPs of the datasets pre- and post-virtual brain segmentation (Figure 3), in the absence of contrast, detectable vessels included large caudal arteries, while small rostral vessels and slow-flowing veins were not visible. Such enhancement is characteristic of the blood flow-dependent TOF-effect in which the magnetization of inflowing blood is refreshed, while slow-flowing blood and static background tissue remain saturated by the repeated radiofrequency (RF) pulse (Beckmann, 2000; Krucker et al., 2004). Gd-micelle CE-MRA, however, demonstrated global vascular enhancement indiscriminate of vessel type and location within the field of view (Figure 3). MIPs following virtual brain segmentation further confirmed that Gd-micelle enhancement provided significant vascular contrast throughout the brain not observed in the absence of contrast (Figure 3). These findings led us to perform the Gd-micelle-enhanced (100 μm)^3^ MRA protocol on mice over 2 years of aging.

### Apparent Cerebral Blood Volumes of Aging Brains and Sub-brain Regions

**Figure 4:**
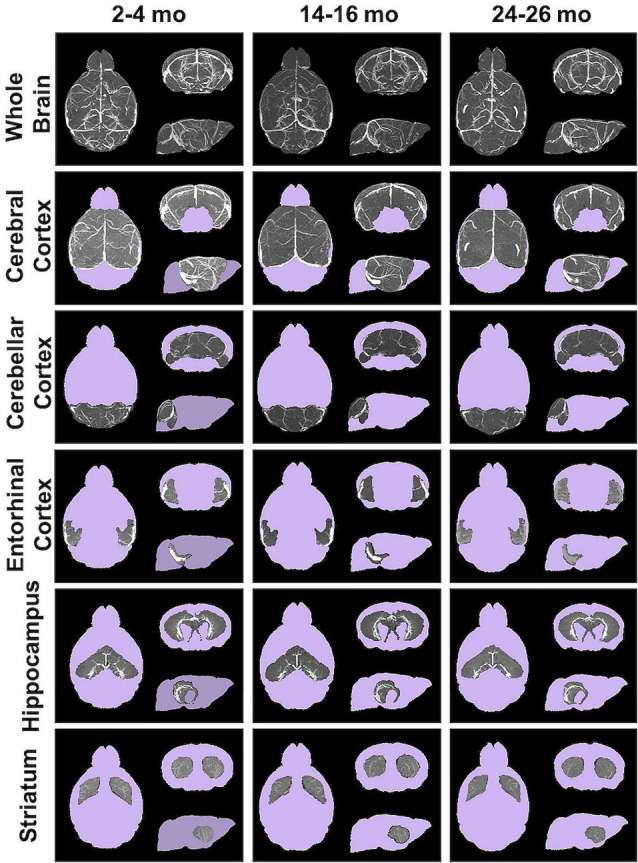
Segmented brain regions of interest from Gd-micelle CE-MRA over aging. Illustrative examples of model brains from mice aged 2–4, 14–16, and 24–26 months demonstrating the virtually segmented whole brain and brain regions. Datasets are displayed as MIPs to illustrate cerebrovascular enhancement.

Angiograms acquired at 2–4 (*N* = 14), 14–16 (*N* = 12), and 24–26 months (*N* = 8) from both C57BL/6NTac mice (Taconic Biosciences, Rensselaer, NY, USA) studied longitudinally and C57BL/6N mice (Charles River Laboratories, Wilmington, MA, USA) were compiled and aligned using automated registration. The brains and brain ROIs were segmented using the Dorr et al. (2008) C57BL/6J mouse brain atlas. Following virtual segmentation, MIPs of the ROIs were used to visualize the cerebrovasculature, without obstruction from surrounding facial vessels. MIPs of each segmented ROI, as demonstrated by a representative brain from each age group, qualitatively demonstrated a decrease in the cerebrovasculature with increasing age, although the effect varied per anatomical region (Figure 4).

**Figure 5:**
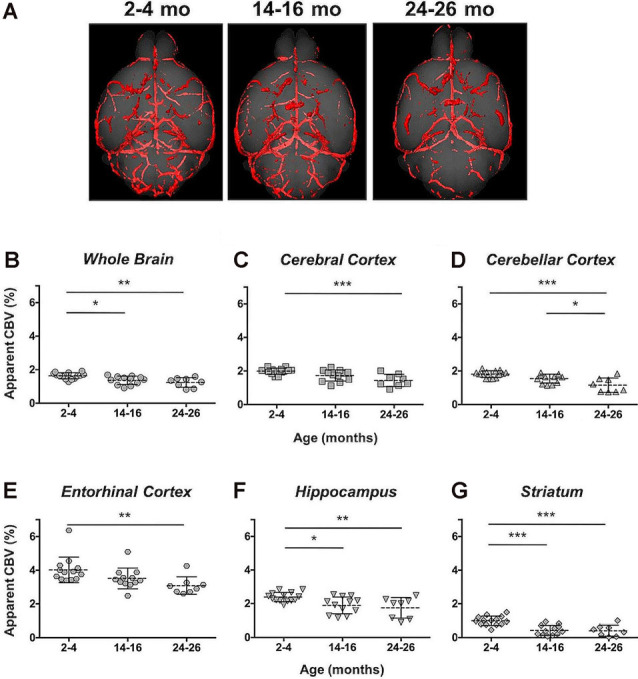
Apparent cerebral blood volume of total C57BL/6 cohort assessed *via* CE-MRA. **(A)** Representative Gd-micelle CE-MR angiograms of murine brains at 2–4, 14–16, and 24–26 months displaying thresholded vessels, shown as MIPs, quantified as the apparent cerebral blood volume (aCBV). The aCBV of all mice studied *via* MRA was calculated and plotted for the **(B)** whole brain, **(C)** cerebral cortex, **(D)** cerebellar cortex, **(E)** entorhinal cortex, **(F)** hippocampus, and **(G)** striatum. Data represent average values and standard deviations. Tukey’s honestly significant difference (HSD) tested was performed to assess the significance of differences between age groups, where *p < 0.05, **p < 0.01, and ***p < 0.001.

The CBV within each brain ROI detected by Gd micelle-enhanced MRA, the aCBV, was quantified following signal-based thresholding. MIPs of the thresholded aCBV in whole brains representative of each age group allowed for a qualitative appreciation of the decrease in the aCBV throughout the 2 years of aging (Figure 5A). The aCBV in each of the six ROIs were quantified for all subjects together, as well as separated by cohorts of C57BL/6NTac subjects and C57BL/6N subjects, acknowledging that phenotypic and genetic differences have been observed among C57BL/6 substrains (Bryant, 2011). The aCBV values for the whole C57BL/6 cohort were determined to be normally distributed in the whole brain and all sub-brain regions. While aging produced region-specific differences, all ROIs examined from the total cohort (C57BL/6NTac and C57BL/6N mice) demonstrated an age-dependent significant decrease in the aCBV by one-way ANOVA (Figures 5B–G, Table 2, Supplementary Table 1) and specifically between 2–4 and 24–26 months brains by Tukey’s HSD test (Figures 5B–G; Table 2). The percent of aCBV loss ranged from 19.95% in the entorhinal cortex to 58.59% in the striatum (Table 2).

**Table 2:** Apparent cerebral blood volumes quantified in the total C57BL/6 cohort.

	Apparent cerebral blood volume (%)					
Age group	Whole brain	Cerebral cortex	Cerebellar cortex	Entorhinal cortex	Hippocampus	Striatum months
2–4	1.64 ± 0.18	1.99 ± 0.18	1.81 ± 0.20	4.02 ± 0.76	2.38 ± 0.27	0.99 ± 0.27
14–16	1.37 ± 0.25	1.73 ± 0.33	1.54 ± 0.27	3.52 ± 0.62	1.89 ± 0.50	0.44 ± 0.30
24–26	1.24 ± 0.29	1.43 ± 0.37	1.15 ± 0.43	3.09 ± 0.53	1.75 ± 0.61	0.41 ± 0.33

*Apparent CBV of brain and brain regions of interest from all mice studied *via* Gd-micelle CE-MRA. Values represent the average volume and standard deviation*.

**Figure 6:**
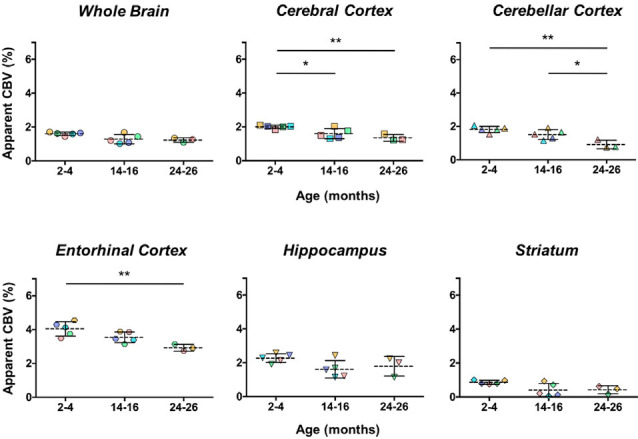
Apparent CBV in C57BL/6NTac mice longitudinally assessed *via* CE-MRA. The apparent CBV was determined in the whole brain and brain regions of longitudinally studied mice. Colors indicate distinct mice aged in-house and imaged over their lifetime. Results of Tukey’s HSD test for significance between age groups are shown, where *p < 0.05 and **p < 0.01.

The aCBV was also separately analyzed for the subset of five C57BL/6NTac mice studied longitudinally through repeat MRA acquisitions at the ages of interest over 2 years (Figure 6, Table 3). However, based on a one-way ANOVA, the hippocampus and striatum showed no significant change in aCBV with age (Figure 6, Table 3, Supplementary Table 1), and Tukey’s HSD test found no significant difference between paired age groups for the whole brain, hippocampus, or striatum (Figure 6, Table 3), possibly in part due to the smaller cohort size and death of two animals before the final imaging session. Notably, the longitudinal investigation highlights the heterogeneity typically described in the aging process (Lockhart and DeCarli, 2014), where specific mice demonstrate steeper declines in the aCBV (Figure 6, blue symbol) than others (Figure 6, yellow symbol; Hagberg and Scheffler, 2013).

**Table 3:** Apparent cerebral blood volumes quantified in C57BL/6NTac mice longitudinally assessed.

	Apparent cerebral blood volume (%)					
Age group	Whole brain	Cerebral cortex	Cerebellar cortex	Entorhinal cortex	Hippocampus	Striatum months
2–4	1.61 ± 0.10	2.00 ± 0.10	1.82 ± 0.19	4.04 ± 0.42	2.28 ± 0.26	0.87 ± 0.12
14–16	1.29 ± 0.28	1.60 ± 0.30	1.51 ± 0.30	3.54 ± 0.32	1.61 ± 0.51	0.42 ± 0.39
24–26	1.24 ± 0.13	1.35 ± 0.21	0.92 ± 0.26	2.93 ± 0.20	1.80 ± 0.58	0.43 ± 0.23

*Apparent CBV of brain and brain regions from mice studied longitudinally via Gd-micelle-enhanced MRA. Values represent the average volume and standard deviation*.

**Figure 7:**
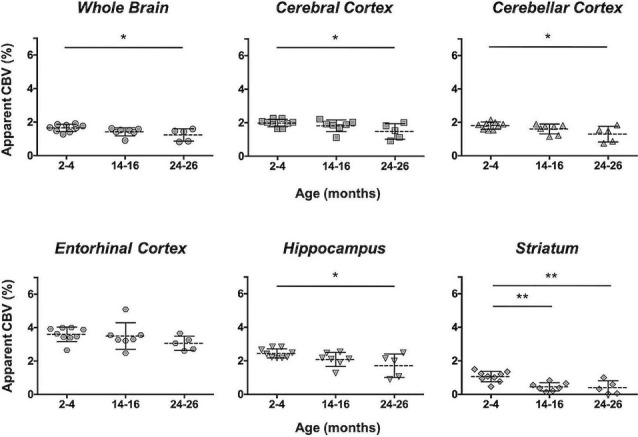
Apparent CBV in C57BL/6N mice assessed *via* CE-MRA. The apparent CBV was determined in the whole brain and brain regions of C57BL/6N mice, excluding the longitudinally studied C57BL/6NTac cohort shown in Figure 6. Results of Tukey’s HSD test for significance between age groups are shown, where *p < 0.05 and **p < 0.01.

Additionally, the cohort of C57BL/N mice was separately studied and revealed significant decreases in the aCBV with age by one-way ANOVA in all regions studied except for the entorhinal cortex (Figure 7, Table 4, Supplementary Table 1). Tukey’s HSD test also found no significant difference between age groups for the entorhinal cortex (Figure 7, Table 4). Notably, based on unpaired two-tailed Student’s *t*-tests performed between the C57BL/6NTac and C57BL/6N cohorts per brain region analyzed, there was no significant difference in the aCBV of C57BL/6NTac and C57BL/6N mice over the 2 years studied.

**Table 4:** Apparent cerebral blood volumes quantified in C57BL/6N mice.

	Apparent cerebral blood volume (%)					
Age group	Whole brain	Cerebral cortex	Cerebellar cortex	Entorhinal cortex	Hippocampus	Striatum months
2–4	1.65 ± 0.22	1.98 ± 0.22	1.80 ± 0.22	3.60 ± 0.43	2.44 ± 0.27	1.06 ± 0.31
14–16	1.42 ± 0.24	1.82 ± 0.35	1.60 ± 0.30	3.50 ± 0.79	2.08 ± 0.42	0.45 ± 0.24
24–26	1.23 ± 0.37	1.48 ± 0.46	1.29 ± 0.47	3.06 ± 0.43	1.71 ± 0.70	0.40 ± 0.41

*Apparent CBV of brain and brain regions from C57BL/6N mice studied *via* Gd-micelle-enhanced MRA. C57BL/6NTac mice studied longitudinally and described in Table 3 are excluded. Values represent the average volume and standard deviation*.

### Volumetric Changes With Aging

**Figure 8:**
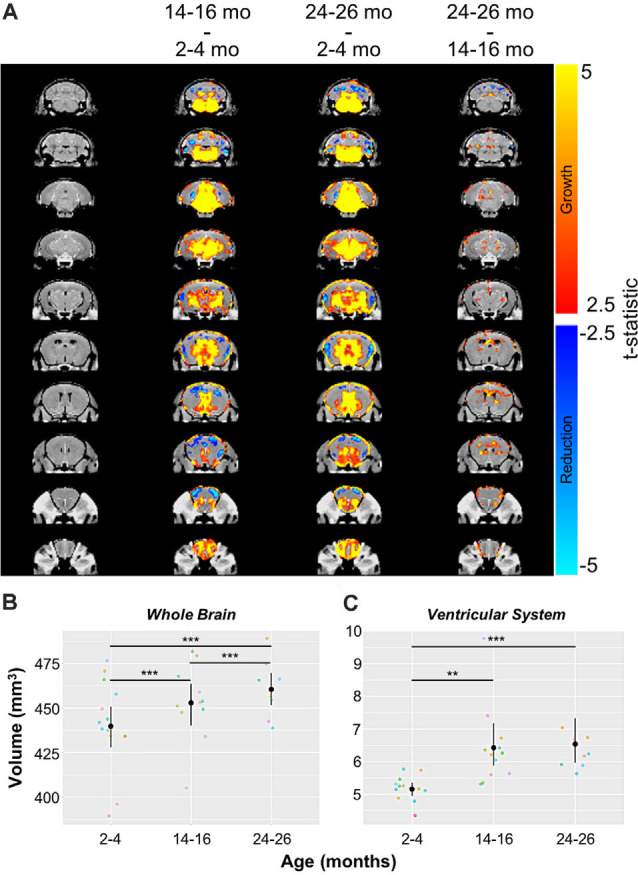
Volumetric changes in the whole brain and ventricular system over aging. **(A)** Deformation maps of global voxel-wise volumetric changes between 2–4 months, 14–16 months, and 24–26 months datasets with significance calculated per voxel with a false discovery rate <5%. Volumetric analyses of **(B)** the whole brain and **(C)** the ventricular system (the compiled lateral ventricles, third ventricle, cerebral aqueduct, and fourth ventricle). Symbol colors in **(B,C)** represent distinct mice and error bars represent the 95% confidence intervals. Separately, one-way analysis of variance (ANOVA) revealed a significant change in volume with age over the 2-year period for both the whole brain and ventricular system (***p < 0.001). Results of Tukey’s HSD *post hoc* test between age groups are shown, where **p < 0.01, and ***p < 0.001.

Automated CE-MRA registration also enabled volumetric comparisons of the brains *via* deformation-based morphometry. Voxel-wise global volumetric differences between the compiled and averaged 14–16 months datasets and 2–4 months datasets predominantly demonstrated significant growth (red-to-yellow) throughout the brain, while smaller regions of significant volumetric reduction (blue-to-cyan) were also observed (Figure 8A). A comparison of 24–26 months brains to 14–16 months brains revealed sporadic areas of brain growth and reduction (Figure 8A), but substantially fewer voxels showed significant differences in the second year of aging. In addition to voxel-wise global comparisons, volumetric differences between the three age groups were also assessed in the virtually segmented whole brain and ventricular system (Figures 8B–D, Table 5), both previously shown to increase in volume in aging C57BL/6 mice (Chen et al., 2011).

**Table 5:** Volumetric changes over aging in the total C57BL/6 cohort.

Whole brain and ventricular volume (mm^3^)			
Region	2–4 months	14–16 months	24–26 months
Whole brain	439.78 (426.76–451.32)	452.86 (441.18–463.70)	460.40 (451.26–469.53)
Ventricular system	5.16 (4.96–5.36)	6.43 (5.88–7.16)	6.54 (5.97–7.30)

*Volumetric changes in the whole brain and ventricular system from deformation-based morphometry of registered brains. Values represent the average volume and the minimum and maximum values with 95% confidence*.

In agreement with the global voxel-wise assessment, a one-way ANOVA revealed a significant change in the whole brain volume with age over the 2-year-period (***p < 0.001), and Tukey’s HSD test revealed statistically significant differences between each age group (***p < 0.001 for each age group comparison; Figure 8B, Table 5). The ventricular system, comprised of the lateral ventricles, the third ventricle, the cerebral aqueduct, and the fourth ventricle, also demonstrated statistically significant growth over 2 years by one-way ANOVA (***p < 0.001; Figure 8C, Table 5). Tukey’s HSD test revealed significant differences between 2–4 and 14–16 months groups (**p < 0.01) and between 2–4 months and 24–26 months groups (***p < 0.001; Figure 8C, Table 5).

### Immunohistochemical Analysis of the Cerebrovascular Density

**Figure 9:**
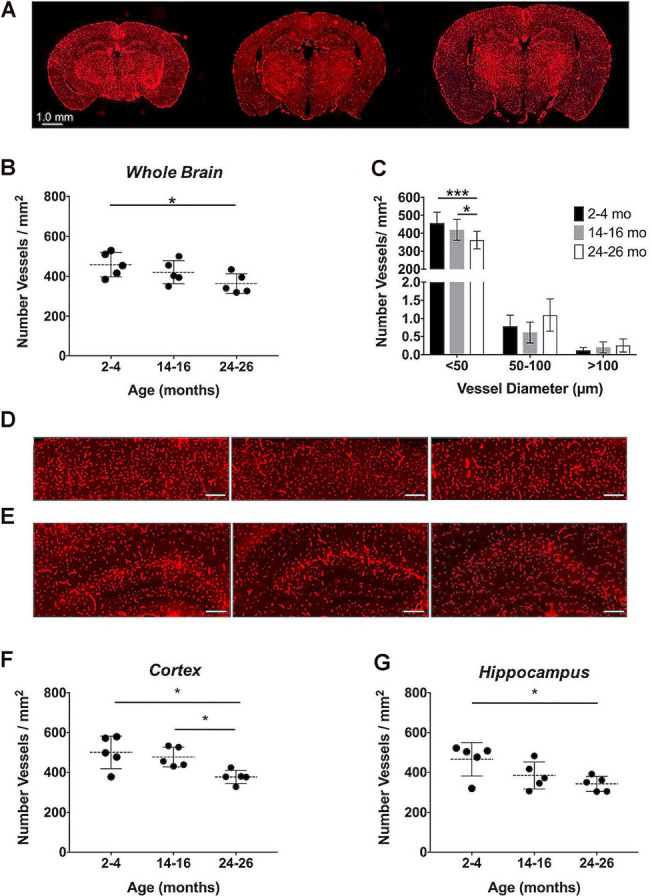
Immunohistochemical quantification of the cerebrovascular density over aging. **(A)** Representative CD31-stained histological sections of mouse brains at 2–4, 14–16, and 24–26 months (left-to-right). Quantification of the cerebrovascular density detected in the entire brain sections **(B)** overall and **(C)** separated by vessel size. Representative sections of the **(D)** cerebral cortex and **(E)** hippocampus at 2–4, 14–16, and 24–26 months (left-to-right), where scale bars indicate 200 μm. Quantifications of the cerebrovascular density in the **(F)** cerebral cortex and **(G)** hippocampus. All data represent the average density and standard deviation (*N* = 5 per age group). Results of Tukey’s HSD test are shown, where *p < 0.05 and ***p < 0.001.

Immunohistochemistry, the gold-standard technique for microvascular visualization (Moy et al., 2013), was employed to assess if a structural loss of vessels, or rarefaction, contributed to the reduction in aCBV detected by Gd-micelle CE-MRA. Brain sections probed for vascular CD31 from each age group (*N* = 5 per age; Figure 9A) were quantified as the number of vessels/mm^2^ throughout the section (Figures 9B,C, Table 6) as well as in the well-defined cerebral cortex and hippocampus (Figures 9D–G, Table 6). Quantification of whole sections demonstrated a reduction in vascular density with age from 458.18 ± 61.28 vessels/mm^2^ at 2–4 months to 362.94 ± 49.34 vessels/mm^2^ at 24–26 months, with *p* = 0.05 by one-way ANOVA and *p* < 0.05 by Tukey’s HSD test between the 2–4 and 24–26 months groups (Figure 9B, Table 6). Additionally, the vessel composition was assessed by size and compared for each age group, with both age (*p < 0.05) and vessel size (****p < 0.0001) significantly contributing to changes in vascular density *via* two-way ANOVA (Figure 9C, Supplementary Figure 1). Furthermore, the density of vessels with diameters <50 μm, characteristic of most vessels detected by IHC, decreased from 456.92 ± 60.62 vessels/mm^2^ at 2–4 months to 419.35 ± 57.91 vessels/mm^2^ at 14–16 months and finally to 361.60 ± 49.03 vessels/mm^2^ at 24–26 months. There was a significant difference between 14–16 and 24–26 months sections (*p < 0.05) and between 2–4 and 24–26 months sections (***p < 0.001) by Tukey’s HSD *post hoc* test. By contrast, vessels with diameters between 50–100 and >100 μm were significantly less detected *via* IHC and showed no statistically significant difference amongst the age groups *via* Tukey’s HSD test, suggesting that the majority of the decrease in density over 2 years was due to a reduction in vessels <50 μm in diameter. However, tissue deformation and shrinkage during histological preparation could affect these measurements (Wehrl et al., 2015).

**Table 6:** Cerebrovascular density in aged brains by immunohistochemical analysis.

Cerebrovascular density (vessels/mm^2^) by CD31-stained immunohistochemistry			
Age group months	Whole brain	Cerebral cortex	Hippocampus
2–4	458.18 ± 61.28	500.60 ± 81.91	465.93 ± 83.71
14–16	420.15 ± 57.96	476.97 ± 49.18	384.32 ± 68.26
24–26	362.94 ± 49.34	377.02 ± 33.91	341.89 ± 38.18

*Quantification of the cerebrovascular density analyzed *via* CD31-probed IHC. Values represent the average density and standard deviation (*N* = 5)*.

The cerebrovascular densities in the cerebral cortex and hippocampus, well-defined within the histological sections, were also quantified in IHC sections. One-way ANOVA tests found statistically significant changes in cerebrovascular density with age across groups in both the cortex and hippocampus (*p < 0.05). The cerebrovascular density of the cerebral cortex (Figure 9D) showed a minor decrease after 1-year aging, but, according to Tukey’s HSD test, there was a significant decrease between 2–4 and 24–26 months age groups from 500.60 ± 81.91 to 377.02 ± 33.91 vessels/mm^2^ (*p < 0.05) and between 14–16 and 24–26 months sections (*p < 0.05; Figure 9F, Table 6). According to Tukey’s HSD test, the hippocampus (Figure 9E) demonstrated a significant decrease in the cerebrovascular density between 2–4 and 24–26 months age groups from 465.93 ± 83.71 to 341.89 ± 38.18 vessels/mm^2^ (*p < 0.05; Figure 9G, Table 6).

## Discussion

The present study investigated the detectable aCBV of normal aging in wild type C57BL/6 mice using the non-destructive and minimally invasive CE-MRA approach throughout a 2-year aging period, accompanied by IHC analysis. To image the cerebrovasculature throughout the murine brain, indiscriminate of vessel type and location, CE-MRA was performed using a self-assembling Gd-micelle blood pool agent synthesized in-house to induce high and sustained *r*_1_ relaxivity within the blood pool, resulting in *T_1_-*weighted vessel contrast throughout the acquisition time needed for high-resolution MRA. This protocol enabled us to acquire MR angiograms for vascular quantification and the monitoring of volumetric changes compared to CD31-probed IHC.

In contrast to commercially available low molecular weight Gd-DTPA (Magnevist), which is extravasated from the vascular space within 30 min, the Gd-micelles remained circulating in the vasculature for at least 2 h, at a 4-fold lower Gd dose, and cleared by 24 h (Figure 2). The significant circulation time of the Gd-micelles is most likely due to both size (Torchilin et al., 2000) and PEGylation (Croy and Kwon, 2006; Nishiyama and Kataoka, 2006; Qiu et al., 2007). With an average 15.63 nm diameter (Table 1), extravasation of the blood pool agent is largely inhibited (Torchilin et al., 2000; Aime and Caravan, 2009) and PEG chains at the micellar surface, contributed by PEG2000-DSPE (Figure 1A), prevent serum protein adsorption and antibody opsonization to further extend circulation time (Croy and Kwon, 2006; Nishiyama and Kataoka, 2006; Qiu et al., 2007). The agent’s large size also contributes to a slower tumbling rate, which effectively increased its relaxivity (Lauffer, 1987, 1991), providing a 3.92-fold increase in *r*_1_ over that of Magnevist per mM Gd (Table 1). With improved relaxation properties, the Gd-micelles was able to be injected at a 4-fold lower Gd dose than recommended for Magnevist (Lewis et al., 2012), reducing the potential risk of Gd-induced toxicity (Grobner and Prischl, 2007), while still providing adequate SNR, 3.19-fold higher than in the absence of contrast (Figure 3), for cerebrovascular detection. Together these results encouraged the use of Gd-micelles as a *T*_1_-shortening blood pool agent for MRA studies of the cerebrovasculature in aging mice.

Automated registration (Friedel et al., 2014) of the MRA datasets to the previously developed C57BL/6J brain atlas (Dorr et al., 2008) compiled images acquired at 2–4, 14–16, and 24–26 mo within the same 3D space, allowing for virtual extraction of the whole brain as well as segmentation of major sub-brain ROIs. A visual assessment of the aligned brains and ROIs presented as MIPs revealed a loss of MRA-detectable vasculature, as demonstrated by model brains representative of each age group (Figure 4). Signal intensity-based thresholding of the cerebrovasculature that fit the criteria of aCBV in the whole brain volume (Figure 5A) revealed a 1.64% ± 0.18% aCBV at 2–4 months (Figure 5B, Table 2), within the 1–4.4% range previously described for the CBV in young adult murine brains (Boero et al., 1999; Heinzer et al., 2006, 2008; Serduc et al., 2006; Chugh et al., 2009; Tsai et al., 2009; Wu et al., 2014; Zhang et al., 2018), including those of the C57BL/6 strain (Chugh et al., 2009; Tsai et al., 2009; Wu et al., 2014; Zhang et al., 2018). Furthermore, the aCBV changes in the whole brain volume equated to an overall 24.39% loss of the aCBV over 2 years aging (Figure 5B, Table 2). While most aging studies focus on vascular quantification in specific regions of the brain, rather than the global cerebrovasculature, this loss is comparable to an *ex vivo* stereological examination of aged rats that demonstrated a 16% loss in global cerebrovasculature (Buchweitz-Milton and Weiss, 1987).

Specific sub-brain regions also demonstrated statistically significant decreases in the aCBV throughout aging (Figures 5C–G, Table 2). The cortex and hippocampus, two of the most widely examined regions for CBV loss in aging (Brown and Thore, 2011), demonstrated a 28.14 and 26.47% aCBV loss over 2 years, respectively (Figures 5C,F, Table 2). Both results are in agreement with previous studies of vascular reductions in the rat cortex (12–43% loss; Burns et al., 1981; Amenta et al., 1995) and hippocampus (20%–49%; Jucker et al., 1990; Amenta et al., 1995). While the smaller subset of mice studied longitudinally demonstrated similar losses in aCBV over aging, these reductions only demonstrated significance within the cerebral cortex, cerebellar cortex, and entorhinal cortex (Figure 6, Table 3), likely an effect of the smaller cohort size. Overall, Gd-micelle CE-MRA with 100 (μm)^3^ resolution detected a decrease in the aCBV throughout the brain ROIs studied; however, improvements in sequence parameters, the use of cryoprobes, or increased Gd-micelle dose, may aid in achieving a higher spatial resolution to resolve vessels <100 μm in diameter, multiple of which may be contained within a single (100 μm)^3^ voxel in the current study.

Deformation-based morphometry revealed regions demonstrating both statistically significant growth and statistically significant reduction, with the majority of the brain showing growth from 2–4 to 14–16 months (Figure 8A). To a lesser extent, although still statistically significant, growth continued between 14–16 and 24–26 months (Figure 8A). Analysis of the whole brain and ventricular system both showed statistically significant increases in volume over the 2-year aging study with a 4.69% increase in whole brain volume and 26.74% increase in the volume of the ventricular system between mice aged 2–4 months and those aged 24–26 months, comparable to the percent enlargement demonstrated by Chen et al. (2011) in the whole brain and ventricles of similarly aged C57BL/6 mice. The percent volume occupied by the ventricles increased from 1.17% of the whole brain at 2–4 months to 1.42% at 14–16 months and 24–26 months (Figures 8B,C, Table 5). Previous aging studies have demonstrated statistically significant whole brain and ventricular growth in wild type mice over 14 months (Maheswaran et al., 2009) and nearly 2 years (Chen et al., 2011), with the relative brain volume occupied by the ventricles enlarging faster during early adulthood, before slowing at older ages. It is hypothesized that increases in ventricular volume beyond that demonstrated in normal aging could serve as a biomarker for Alzheimer’s disease and its progression (Nestor et al., 2008; Chen et al., 2011). As demonstrated, CE-MRA is capable of simultaneously providing information on the volumetric changes of both the CBV and anatomical brain regions, supporting its use in exposing the differences in normal brain aging and disease.

IHC, probing for the endothelial cell marker CD31, demonstrated a statistically significant decrease in the cerebrovascular density over 2 years, with an overall 20.79% decrease (Figures 9A,B, Table 6). Although CE-MRA and IHC quantified the vasculature differently, percent volume (mm^3^/mm^3^) for CE-MRA and density (vessels/mm^2^) for IHC, each with distinct limitations, the detected 20.79% percent decrease in cerebrovascular density by IHC was in good agreement with the 24.39% decrease in aCBV over 2 years. An evaluation of the vessel sizes detected that the major population of vessels, with diameters <50 μm, showed a significant reduction in density over 2 years (Figure 9C), supporting the well-described microvascular, particularly capillary, rarefaction detected in human and rat brains (Riddle et al., 2003; Brown and Thore, 2011). Cortical and hippocampal analyses by IHC also demonstrated significant decreases in vessel density over 2 years of aging (Figures 9D–G, Table 6). The 24.69% loss in vascular density that was detected in the cortex by IHC from 2–4 to 24–26 months (Figures 9D,F) was comparable to the 28.14% loss determined in the cortex by CE-MRA (Figure 5C, Table 2). Furthermore, these values are within the range of vascular loss described for rodent brains over aging (12–43%; Brown and Thore, 2011), but are moderately higher than the 19.26 ± 6.7% decrease recently described by Murugesan et al. (2012) in aging male C57BL/6J mice using *ex vivo* confocal microscopy between 6 and 24 months. IHC of the hippocampus demonstrated a 26.62% loss in vessel density over 2 years (Figures 9E,G, Table 6), compared to an overall 26.47% decrease detected in the hippocampus by CE-MRA (Figure 5F, Table 2). Similarly, these values were within the range of vessel loss described in aging rats (20–49%; Brown and Thore, 2011) and were also in good agreement with the hippocampal loss detected by Murugesan et al. (2012) of 26.38 ± 5.63% in C57BL/6J mice.

While CE-MRA and IHC maintain vastly different capacities for resolving the cerebrovasculature, these techniques can be used in a complementary manner to monitor cerebrovascular alteration. Here, both methods have revealed a significant loss in vascularity in the aging C57BL/6 female mouse brain over a 2-year period. Differences between the two methods are contributed by MRA’s lower resolution, whereby the current MRA protocol is capable of detecting, but not resolving, the <50 μm-diameter vessels largely identified *via* IHC. The IHC-determined loss in the number of vessels/mm^2^ within whole brain sections, as well as in cortical and hippocampal regions, suggests that a structural loss in vascularity contributed significantly to the reduction in aCBV detected *via* CE-MRA.

Differences between the degree of cerebrovascular rarefaction described herein and that reported by Murugesan et al. (2012) may be due to the two studies’ differing imaging modalities (MRA vs. confocal microscopy) and the nature of the tissue (*in vivo* vs. *ex vivo*). However, sex differences may also play a major role in brain aging. By limiting our study to aging female subjects, it is unknown if aging male C57BL/6 mice would have demonstrated a comparable loss of cerebrovasculature using our micelle-enhanced MRA methodology. Notably, previous studies of C57BL/6 mice have found that, compared to their aging male counterparts, aging female mice demonstrate a greater cognitive decline (Benice et al., 2006). An earlier onset for age-related hippocampal genetic alterations in the same mouse strain was also found in females that resulted in decreased bioenergetic metabolism and increased amyloid dyshomeostasis (Zhao et al., 2016). Considering these findings, the significant loss of cerebrovasculature in this female C57BL/6 cohort may serve as yet another characteristic of brain aging to which female mice may be more susceptible. Therefore, the use of an entirely female cohort could contribute to the greater vascular loss detected in the current study compared to that determined by Murugesan et al. (2012) for an all-male C57BL/6 cohort. Still, a single study of both sexes under identical conditions is needed to elucidate such differences. Furthermore, our results support previous research in demonstrating the profound impact that aging has on the brains of female C57BL/6 mice. How this translates to aging humans and predisposition to age-dependent conditions including Alzheimer’s disease is still under investigation. Dementia and Alzheimer’s disease have shown a higher prevalence in XX chromosome-harboring women over XY-harboring men (Beam et al., 2018). However, recent studies find that XX-women may have metabolically younger brains (Goyal et al., 2019) and demonstrate a slower rate of regional brain volume loss compared to age-matched XY-men (Armstrong et al., 2019). The evident complexity of brain aging and how it is impacted by variables including sex and co-morbidities is a further reason for conducting longitudinal studies in aging murine models as they may identify associated genetic, functional, and anatomic changes.

In addition to using this methodology to investigate age-matched sex differences, it can also be employed for qualitative and quantitative assessments of cerebrovascular alterations in neurovascular diseases and response to therapeutic interventions. The rapid recovery time following the femoral injection of the micellar contrast agent and clearance within 24 h allows our approach to be repeatable. Therefore, our MRA method is particularly well-suited for longitudinal studies aiming to track and localize the progression of regional vascular changes in angiopathies and neurodegenerative diseases. Additionally, this noninvasive and non-ionizing imaging technique can be equally valuable to monitor the response to therapy at a multitude of time points as well as to assess the variability amongst individual subjects. This technique is also applicable for single time point cross-sectional studies followed by IHC 24 h later.

In considering repeated studies, our choice of femoral cannulation was motivated by our need to control the precise dose and rate of infusion of the micellar blood pool agent to ensure reproducibility and prevent mis-administration typically associated with tail injection (Groman and Reinhardt, 2004). Hence, femoral cannulation would not be appropriate for daily examination. Instead, an indwelling catheter system such as those employed in repeat murine blood sampling (Park et al., 2018) could be considered. Alternatively, tail vein injection may be considered when performed by highly skilled researchers, enabling frequent injections. Animal exposure to anesthesia, such as isoflurane employed in our study, is another limiting factor. Repeated exposure to isoflurane, while generally considered a safe anesthetic option, has been reported to cause anxiety, motor deficits, altered white matter integrity (Bajwa et al., 2019), and oxidative stress (Berkowitz et al., 2020). The qualitative micelle pharmacokinetic characterization performed in Figure 2 illustrates the tradeoff between effective vascular detection and complete micelle washout between imaging sessions. The reduced dose of 0.75 μmol Gd/30 g Gd-micelle enabled full clearance within 24 h and reduced the risk of Gd-induced toxicity (Grobner and Prischl, 2007) while providing remarkable vascular enhancement.

CE-MRA offers the advantage of indiscriminate whole head detection and visualization of both the arterial and venous blood volumes regardless of flow. In comparison, TOF-MRA typically limits the detection to arteries within a reduced FOV, which are governed by the inflowing strength of proximal blood. This in turn predominantly excludes the venous component (Axel, 1984; Nishimura, 1990). On the other hand, CE-MRA prevents an immediate understanding of whether cerebrovascular changes predominate in arteries or veins. In this case, the alignment of acquired images to cerebral vascular atlases, such as those described by Dorr et al. (2007) and Xiong et al. (2017), will help delineate the venous and arterial compartments and permit their respective quantification. This additional step may facilitate the examination of mouse models of cardiovascular diseases, including heart failure associated with increased cerebral venous pressure, small vessel damage, and blood-brain barrier disruption (Fulop et al., 2019).

## Conclusion

We have observed a significant loss in cerebrovasculature over 2 years of aging in the C57BL/6 mouse strain using both *in vivo* whole head CE-MRA and IHC-probed histological analysis. While the majority of our knowledge regarding the cerebrovasculature is the result of *ex vivo* 2D studies, the use of *in vivo* whole-brain analyses, such as the CE-MRA technique described here, can improve our understanding of the global murine cerebrovasculature, its variability amongst subjects and strains, and its changes during aging. The (100 μm)^3^ resolution employed is significantly lower than that of histological examination. However, CE-MRA still provided minimally invasive *in vivo* assessment of the cerebrovascular morphology and detectable aCBV with coverage of the entire murine head. Due to its non-destructive and non-to-minimally invasive nature, this technique allowed for both single time point and longitudinal studies in aging subjects to illustrate significant, albeit variable, loss of CBV throughout the aging murine brain along with enlargement of the whole brain and ventricular volumes. Together, the changes detected by CE-MRA and IHC suggest that the vascular rarefaction widely-described in aging human and rat brains is also present in the most widely studied inbred mouse strain (Bryant, 2011). Age-dependent vascular rarefaction in wild type C57BL/6 mice should, therefore, be accounted for when using this popular strain for studies of age-dependent diseases of the cerebrovasculature.

## Data Availability Statement

The raw data supporting the conclusions of this article will be made available by the authors, without undue reservation.

## Ethics Statement

The animal study was reviewed and approved by NYU Grossman School of Medicine’s Institutional Animal Care and Use Committee (IACUC).

## Author Contributions

LH: conceptualization, methodology, validation, formal analysis, investigation, data curation, writing—original draft, and visualization. DH: methodology, software, validation, and data curation. LC: methodology, validation, investigation, resources, data curation, and writing—review & editing. TW: methodology, and writing—review & editing. MS: methodology, resources, writing—review & editing, and funding acquisition. YW: conceptualization, methodology, software, resources, writing—review & editing, supervision, project administration, and funding acquisition. All authors contributed to the article and approved the submitted version.

## Conflict of Interest

The authors declare that the research was conducted in the absence of any commercial or financial relationships that could be construed as a potential conflict of interest.
