# Supplementary material for: Detection of Cerebrovascular Loss in the Normal Aging C57BL/6 Mouse Brain Using in vivo Contrast-Enhanced Magnetic Resonance Angiography
Source: Front Aging Neurosci. 2020 Oct 20;12:585218. doi: 10.3389/fnagi.2020.585218 (PMC7606987; doi:10.3389/fnagi.2020.585218)
Supplement: Supplementary file 1 [file Data_Sheet_1.pdf]

Supplementary Data

Figures (1) and Tables (1)

Detection of Cerebrovascular Loss in the Normal Aging C57BL/6 Mouse Brain Using *in vivo* Contrast-Enhanced Magnetic Resonance Angiography

Lindsay K. Hil [1,2,3,4], Dung Minh Hoang [2,3], Luis A. Chiriboga [5], Thomas Wisniewski [5,6,7], Martin J. Sadowski [6,7,8], Youssef Z. Wadghiri [2,3]*

[1] Department of Chemical and Biomolecular Engineering, NYU Tandon School of Engineering, Brooklyn, NY, United States
[2] Center for Advanced Imaging Innovation and Research (CAI²R), Department of Radiology, NYU School of Medicine, New York, NY, United States
[3] Bernard and Irene Schwartz Center for Biomedical Imaging, Department of Radiology, NYU School of Medicine, New York, NY, United States
[4] Department of Biomedical Engineering, SUNY Downstate Medical Center, Brooklyn, NY, United States
[5] Department of Pathology, New York University School of Medicine, New York, NY, United States
[6] Department of Neurology, NYU School of Medicine, New York, NY, United States
[7] Department of Psychiatry, NYU School of Medicine, New York, NY, United States
[8] Department of Biochemistry & Molecular Pharmacology, NYU School of Medicine, New York, NY, United States

* Corresponding Author
Email: wadghiri@med.nyu.edu

ORCID:

Lindsay K. Hill	: 0000-0002-1833-8574
Dung Minh Hoang	: 0000-0001-7613-7167
Luis A. Chiriboga	: 0000-0002-2028-6873
Thomas Wisniewski	: 0000-0002-3379-8966
Martin J. Sadowski	: 0000-0002-3830-1779
Youssef Z. Wadghiri	: 0000-0001-7175-9397

Supplementary Data

Significance in Apparent Cerebrovascular Volume (%) Changes with Age in C57BL/6 Mice by one-way ANOVA

Subject Group	Whole Brain	Cerebral Cortex	Cerebellar Cortex	Entorhinal Cortex	Hippocampus	Striatum
**Total C57BL/6 Cohort**	***	***	***	**	**	****
**C57BL/6NTac**	*	**	**	**	ns	ns
**C57BL/6N**	*	*	*	ns	*	**

Supplementary Table 1 Significance in apparent cerebral blood volume changes with age quantified in C57BL/6 mice via one-way ANOVA. Summary of the significance by one-way ANOVA in all subject groups per brain region analyzed. One-way ANOVA significance results are summarized as ns= no significance, * $p < 0.05$, ** $p < 0.01$, *** $p < 0.001$, and **** $p < 0.0001$.

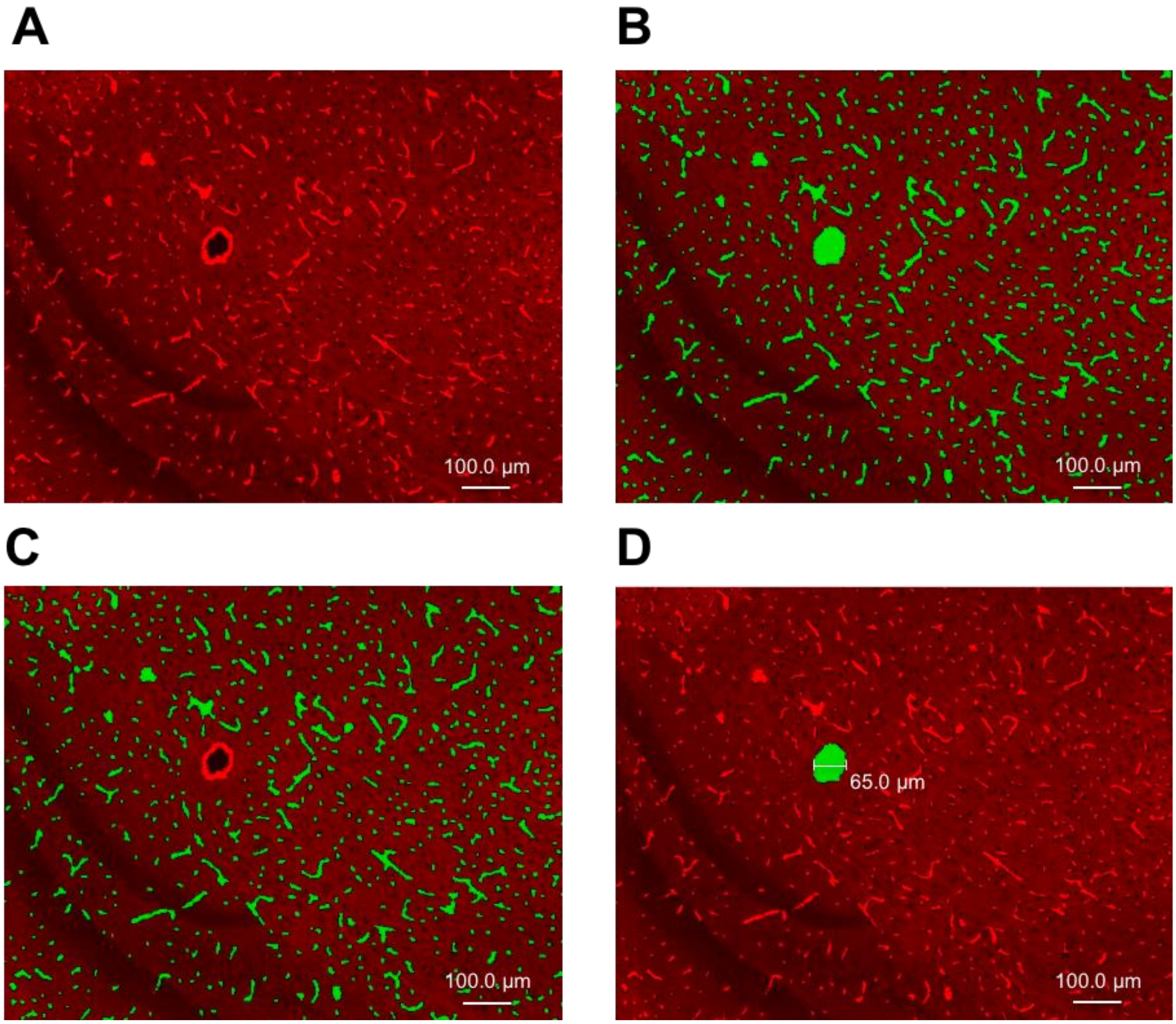

Supplementary Figure 1 CD31-stained immunohistochemical analysis of the cerebrovasculature. **(A)** Example of a 2-4 month brain section probed for CD31 using immunohistochemistry to quantify the cerebrovascular density and vessel size. **(B)** Cerebrovasculature for quantification was thresholded via red fluorescence intensity, with thresholded vessels highlighted in green. Vessel sizing by thresholding the minor axes was conducted to evaluate vessels with diameters **(C)** < 50 µm, **(D)** 50-100 µm, and > 100 µm.
